# The extended phenotypes of marine symbioses: ecological and evolutionary consequences of intraspecific genetic diversity in coral–algal associations

**DOI:** 10.3389/fmicb.2014.00445

**Published:** 2014-08-25

**Authors:** John E. Parkinson, Iliana B. Baums

**Affiliations:** Department of Biology, The Pennsylvania State University, University ParkPA, USA

**Keywords:** coral, genotype interactions, intraspecific diversity, mutualism, *Symbiodinium*

## Abstract

Reef-building corals owe much of their success to a symbiosis with dinoflagellate microalgae in the genus *Symbiodinium*. In this association, the performance of each organism is tied to that of its partner, and together the partners form a holobiont that can be subject to selection. Climate change affects coral reefs, which are declining globally as a result. Yet the extent to which coral holobionts will be able to acclimate or evolve to handle climate change and other stressors remains unclear. Selection acts on individuals and evidence from terrestrial systems demonstrates that intraspecific genetic diversity plays a significant role in symbiosis ecology and evolution. However, we have a limited understanding of the effects of such diversity in corals. As molecular methods have advanced, so too has our recognition of the taxonomic and functional diversity of holobiont partners. Resolving the major components of the holobiont to the level of the individual will help us assess the importance of intraspecific diversity and partner interactions in coral–algal symbioses. Here, we hypothesize that unique combinations of coral and algal individuals yield functional diversity that affects not only the ecology and evolution of the coral holobiont, but associated communities as well. Our synthesis is derived from reviewing existing evidence and presenting novel data. By incorporating the effects of holobiont extended phenotypes into predictive models, we may refine our understanding of the evolutionary trajectory of corals and reef communities responding to climate change.

## INTRODUCTION

Fundamentally, evolution by way of natural selection acts on functional variation among individuals within a species (Darwin, 1859; Fisher, 1930). When the success of two (or more) organisms are linked, such as among mutualistic symbiotic partners, variation within one species interacts with the variation in the other, as well as with the environment (Thompson, 2005; Warren and Bradford, 2014), potentially driving direct and indirect evolutionary interactions (Wootton, 1994; Rowntree et al., 2014). Thus, the adaptive capacity of symbiotic organisms will be underestimated when intraspecific variation is not accounted for (Fisher, 1930). The increasing scale of reef degradation has called into question the ability of coral–algal symbioses to acclimate or evolve to deal with a changing world (Lasker and Coffroth, 1999; Glynn et al., 2001; Hoegh-Guldberg et al., 2002; Reshef et al., 2006; Brown and Cossins, 2011; Barshis et al., 2013). Acclimation occurs over the course of an organism’s lifetime, while evolution takes place over generations; the time frame for both processes can overlap when evolution is particularly rapid (Hairston et al., 2005). Despite the fact that host and symbiont genomes are often decoupled each generation, coevolution clearly occurs (Thornhill et al., 2014). Current forecasts of reef perseverance do not explicitly incorporate the effects of intraspecific diversity driving coevolution among coral–algal partners because such effects have rarely been assessed.

Classically, biodiversity has been measured at the species level, and such diversity has generally had positive effects on higher-order community diversity, function, and resilience (Balvanera et al., 2006). Modern molecular techniques are revolutionizing species delineation in coral holobionts. Using genetic and complementary phenetic evidence, many traditional host species designations and higher-order relationships are being reevaluated (Fukami et al., 2004, 2008; Huang et al., 2011; Pinzon and LaJeunesse, 2011; Budd et al., 2012; Keshavmurthy et al., 2013). Microalgae (including *Symbiodinium*) are likewise receiving renewed taxonomic attention emphasizing molecular data (LaJeunesse et al., 2012, 2014; Jeong et al., 2014; Leliaert et al., 2014).

More recently, intraspecific diversity has been revealed to be just as important (in some cases, more important) than interspecific diversity in explaining variation in associated community traits (Hughes et al., 2008). For example, the diversity, richness, and abundance of arthropods on trees are better explained by the number of tree genotypes than tree species diversity (Shuster et al., 2006; Whitham et al., 2006). However, similar investigation is lacking for corals and their microalgae. Few studies have addressed whether genotype diversity of a coral species affects the diversity of its symbiont community or other associated invertebrates and vertebrates. This is partly because the resolution of species (let alone individuals) in the coral holobiont has been contentious (Stat et al., 2012). Within a given coral species, morphologically distinct colonies can be genetically identical owing to phenotypic plasticity among asexual fragments (Highsmith, 1982; Todd, 2008), while genetically disparate colonies may share striking resemblance (e.g., Pinzon and LaJeunesse, 2011). All *Symbiodinium* species and cell lines look superficially similar even under high magnification (LaJeunesse, 2001). Without high-resolution genetic markers, intraspecific effects on the ecology and evolution of coral–algal symbioses have been difficult to quantify accurately.

Population genetic microsatellite markers are increasingly used to study both scleractinian hard corals (Lopez et al., 1999; Maier et al., 2001; Magalon et al., 2004; Severance et al., 2004; Baums et al., 2005a, 2009; Underwood et al., 2006; Mangubhai et al., 2007; van Oppen et al., 2007; Isomura and Hidaka, 2008; Starger et al., 2008; Andras and Rypien, 2009; Wang et al., 2009; Concepcion et al., 2010; Polato et al., 2010; Banguera-Hinestroza et al., 2013; Chen et al., 2013; Davies et al., 2013) and *Symbiodinium* (Santos and Coffroth, 2003; Magalon et al., 2004; Pettay and LaJeunesse, 2007, 2009; Bay et al., 2009; Howells et al., 2009; Kirk et al., 2009; Andras et al., 2011; Pinzon et al., 2011; Wham et al., 2011, 2014). Armed with such markers, it is now possible to sample a single coral colony and determine not only its host and symbiont species compositions, but also to resolve unique multilocus genotypes (i.e., individuals) within each species. However, only rarely have both host and symbiont genotype composition been analyzed in concert (Andras et al., 2011, 2013; Pettay et al., 2011; Pettay and LaJeunesse, 2013; Thornhill et al., 2013; Baums et al., 2014; Prada et al., 2014b). So far this has only been done in a general population survey context, with most evidence suggesting that the genetic structuring of the host and the symbiont are not the same (e.g., Baums et al., 2014). No studies have manipulated host-symbiont pairings to examine genotype level interspecific interactions while unambiguously resolving both partners. Such work is routine in the study of terrestrial mutualisms, but represents a new frontier in the marine realm.

Researchers now stand poised to answer previously intractable questions about the nature of coral–algal symbioses. In this review, we argue that intraspecific diversity is an important component shaping interspecific interactions within a holobiont, and that such interactions may influence the evolutionary trajectory of reef ecosystems faced with a changing climate. We have four major goals: (i) to briefly review the role of intraspecific diversity in other systems, (ii) to describe what we currently know about intraspecific diversity in coral hosts and algal symbionts, (iii) to present preliminary data illustrating the potential extent of functional intraspecific diversity in coral–algal systems, and (iv) to identify research questions and methodologies that will shed further light on this understudied component of marine microbial symbiosis ecology. We posit two central, testable hypotheses: (i) genotypic interactions between coral hosts and algal symbionts influence functional diversity and therefore evolutionary capacity in coral holobionts, and (ii) intraspecific diversity among corals affects reef community function. Dawkins (1982) introduced the concept of “extended phenotypes” to incorporate the indirect effects of genes on the environment independent of the individual bodies in which they reside. In this framework, unique combinations of coral and *Symbiodinium* individuals might be thought of as holobionts with unique extended phenotypes that may shape reef community dynamics.

## SIGNIFICANCE OF INTRASPECIFIC FUNCTIONAL DIVERSITY IN OTHER SYSTEMS

The importance of genotypic diversity (i.e., the number of distinct multilocus genotypes) among symbiotic partners is apparent in terrestrial systems, where genotype level resolution has been used in manipulative experiments for years. An illustrative example is the association between plants and arbuscular mycorrhizal fungi (AMFs). These fungi penetrate vascular plant roots, transmitting nutrients from the surrounding soil to the host. AMFs are obligate symbionts—they cannot survive without a host plant. Numerous studies have recorded symbiont genotype effects on host performance (and vice versa; reviewed by Johnson et al., 2012). For instance, Koch et al. (2006) inoculated clonal carrot roots with genetically distinct AMFs belonging to the single species *Glomus intraradices*; host root growth varied with symbiont genotype. Munkvold et al. (2004) monitored host and symbiont growth among holobionts composed of distinct genotype pairings; growth varied depending on intraspecific partner combinations. Scheublin et al. (2007) found that intraspecific symbiont identity affected the outcome of competitive interactions between the host and other plant species. Similar effects are found in other systems. Among genetically identical host clones of pea aphids, pathogen resistance was conferred to different degrees by distinct strains of a facultative bacterial symbiont species (Lukasik et al., 2013b). Conversely, host pathogen resistance and fecundity varied among host genotypes associating with a clonal symbiont (Lukasik et al., 2013a). These examples highlight that intraspecific diversity among holobiont partners can be high and drive complex interactive effects that mediate holobiont fitness in multiple ways. The same is likely true in coral–algal systems.

The effects of host-symbiont pairings are reflected not only in growth, competitive interactions, pathogen resistance, and fitness, but also in gene expression patterns. Heath et al. (2012) explored the molecular underpinnings of partner interactions by partitioning genetic variation in plant and AMF transcriptomes into additive and interactive effects. The authors found that interactions between plant and AMF genotypes drove symbiont gene expression changes and transitioned host transcription from a nuclear dominated profile (i.e., basic housekeeping) to a plasmid dominated profile (i.e., nitrogen fixation). These polymorphisms altered access to nitrogen fixation, the chief benefit of symbiosis to the plant and a determinant of host reproductive fitness. When the fitness of one species is influenced by the genotype of its symbiotic partner, coevolution is possible (Thompson, 2005; Wade, 2007). Fitness and expression differences among distinct holobionts exemplify natural variation available to coevolutionary selection (Heath et al., 2012). Evolutionary innovation can arise from transcriptional variation in response to short- and long-term stress (Lopez-Maury et al., 2008), and such variation has been described in marine organisms responding to selective pressures associated with climate change, including temperature (e.g., DeSalvo et al., 2010; Barshis et al., 2013; Polato et al., 2013) and acidification (Pespeni et al., 2013). In the coral–algal system, genetically determined expression differences among holobionts responding to stress might be subject to natural selection and lead to adaptation.

Increasingly, diversity below the species level is recognized to be an important force shaping community dynamics, particularly among ecosystem engineers (Whitham et al., 2006; Bolnick et al., 2011). In pea aphid studies, symbiont genotype affected the extent of pathogen sporulation in dead hosts, which likely altered community dynamics by limiting or expanding the exposure of other aphids to the fungus (Lukasik et al., 2013a,b). In the Pacific Northwest, locally derived leaf litter from red alder trees (*Alnus rubra*) decomposed more rapidly than litter derived from trees at other riparian zones, indicating intraspecific variants might drive community-level changes to ecosystem flux (Jackrel and Wootton, 2013). In poplar trees (*Populus* sp.), plant genotype was shown to explain three times as much variation in associated arthropod communities as species level differences (Shuster et al., 2006). Similarly, soil microbial community composition was driven largely by intraspecific genotype (Schweitzer et al., 2008). For the marine eelgrass (*Zostera marina*), genotypically diverse beds were more resistant to disturbance by grazing geese, as were their associated invertebrate fauna (Hughes and Stachowicz, 2004). Intraspecific diversity improved not only seagrass biomass and density but also epifaunal abundance over the course of a warm water temperature anomaly (Reusch et al., 2005). Thus, genotypic diversity in seagrasses has both first-order effects on species resistance and/or resilience as well as second-order effects on ecosystem function. Corals are also marine ecosystem engineers; similar second-order effects may have a profound influence on reef function.

In summary, results from terrestrial studies suggest by extension that intraspecific variation among coral holobionts has the potential to scale up to influence the diversity, resilience, and function of entire reef ecosystem, including associated microbes, alga, invertebrates, and vertebrates. The critical first step in all future studies of intraspecific diversity will be establishing the individual identities of each coral colony and *Symbiodinium* strain under investigation.

## DEFINING CORAL–ALGAL DIVERSITY

The coral holobiont is composed of more than just the host and *Symbiodinium*. Within host tissues, additional symbionts may include apicomplexa (Toller et al., 2002; Kirk et al., 2013a,b), nitrogen-fixing cyanobacteria (Lesser et al., 2004), other bacteria (Rohwer et al., 2002), viruses (Wilson et al., 2005), archaea (Kellogg, 2004; Wegley et al., 2004), and cell-associated microbial aggregates (Work and Aeby, 2014), not to forget organisms found in the host skeletal structure such as endolithic algae (Odum and Odum, 1955; Shashar and Stambler, 1992) and fungi (Le Campion-Alsumard et al., 1995; Bentis et al., 2000). The partner for which the most data are available and for which the role in the symbiosis is most clearly understood is *Symbiodinium*; we therefore use the term “symbiont” to refer only to *Symbiodinium* in this review.

When it was first described, taxonomic diversity among *Symbiodinium* was assumed to be low (Freudenthal, 1962; Taylor, 1984). Over time, it was recognized that the genus included many different species based on various morphological, physiological, and early genetic data (Schoenberg and Trench, 1980a,b,c). Molecular diversity in the group achieved more recognition when *Symbiodinium* were divided into low-resolution clades based on rDNA (Rowan and Powers, 1992), and some corals were found to associate with members of different symbiont clades simultaneously (Rowan et al., 1997). At the time, it was acknowledged that the genetic distances between clades were similar to those observed among different genera and even families of dinoflagellates—an observation borne out by more recent molecular analyses (Stern et al., 2010; Ladner et al., 2012). Higher resolution was achieved by dividing *Symbiodinium* into subcladal “types” using hypervariable regions of nuclear and chloroplast rDNA markers (LaJeunesse, 2001, 2002; Santos et al., 2003a). Now, a suite of hierarchical molecular markers and population genetic data are being used to define precise species boundaries and refine *Symbiodinium* taxonomy (LaJeunesse et al., 2012, 2014; Jeong et al., 2014). Though it has yet to be physically observed, overwhelming molecular evidence indicates that *Symbiodinium* engage in sex at some frequency in the wild, either within the coral habitat or in the external environment (Baillie et al., 2000; LaJeunesse, 2001; Santos et al., 2004; Sampayo et al., 2009; Pettay et al., 2011; Baums et al., 2014; Chi et al., 2014; Thornhill et al., 2014). Sympatric symbionts found in distinct colonies of the same host species in the same environments exhibit diagnostic microsatellite allele frequencies, revealing genetic recombination within but not between groups (LaJeunesse et al., 2014). This satisfies the biological species concept, demonstrating that molecular data can be used to consistently delimit species boundaries in *Symbiodinium*—a necessity for investigating intraspecific diversity.

Similar molecular data have been used to resolve coral host species, which feature the added complication of introgressive hybridization among closely related taxa (Ladner and Palumbi, 2012). Often, current taxonomic designations based on morphological characteristics are at odds with genetic evidence. For example, the entity designated *Stylophora pistillata* was recently determined to be composed of at least four species based on cytochrome oxidase I sequencing (Keshavmurthy et al., 2013), while multiple markers suggest that three of the Caribbean poritid morphospecies (*Porites divaricata, P. furcata,* and *P. porites*) should be collapsed into one entity (Prada et al., 2014a). Even within a single genus, molecular data indicate some lineages should be lumped while others should be split (Pinzon et al., 2013). Unlike *Symbiodinium*, it will be easier to combine data from experimental crosses, morphological assessments, and genetic sequencing to resolve coral species (Budd et al., 2010, 2012). Proper species identification is critical when designing experiments to understand coral evolution. Failure to recognize that colonies belong to distinct species when collecting population genetic data can produce misleading signatures of structure and hybridization (Combosch et al., 2008; Combosch and Vollmer, 2011). Failure to recognize cryptic species can also mask important differences in ecological interactions and population dynamics (Boulay et al., 2014). Once coral species boundaries are established, it then becomes possible to assess functional diversity among individuals within species.

Biologically, the notion of an individual is difficult to define in corals. On one level, there is the smallest physical unit representing the organism’s genome (the polyp). On another, there are units of contiguous tissue that connect multiple clonal polyps (the colony). In macro-scale contexts, these colonies are the ecologically significant units on a reef. Sometimes, physically separated colonies are clones (i.e., share the same genome), whereas others are genetically distinct. Throughout this review, when attributed to a given organism, we use the term “genotype” to refer to the concept of genome identity within a species (that is, genetically distinct individuals). All coral colonies that share an identical genome together comprise a “genet,” with each member colony referred to as a “ramet.” Coral genotypic diversity thus refers to the number of distinct genets on a reef.*Symbiodinium* are also capable of both clonal and sexual propagation, but their unicellular nature requires that we use different terminology than corals. A single *Symbiodinium* cell contains one genome and functions independently of all others cells. When residing within host cells, *Symbiodinium* typically reproduce asexually and generate homogenous populations of cells derived from a single ancestor. We use the term “strain” to refer to this physical collection of clonal symbiont cells hosted within a coral colony. In contrast, sexual reproduction leads to new strains. Multiple *Symbiodinium* strains may be present within the habitat provided by a single coral colony, and multiple strains from either a single or many species may be present.

**FIGURE 1:**
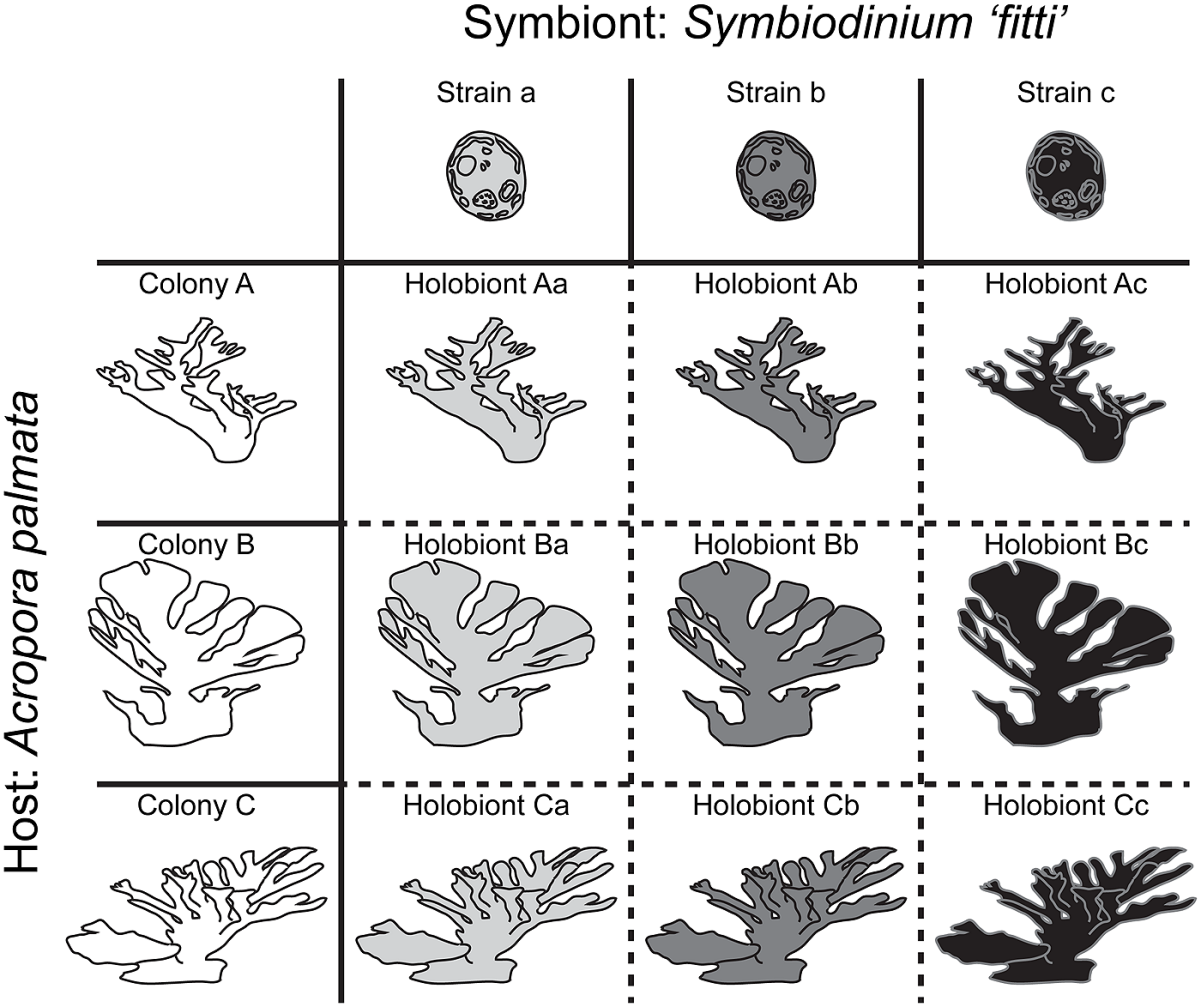
**Diagram showing how coral–algal holobionts represent unique pairings of partner genotypes in the *Acropora palmata–Symbiodinium “fitti”* association.** Host genotype (colony) is indicated by shape; symbiont genotype (strain) is indicated by color. In this association, the host:symbiont genotype ratio is one:one in most colonies. Typically, >99% of each colony’s symbiont population is composed of clonal cells representing a single genotype (that is, one strain).

It has become clear that in many coral–algal symbioses, individual host colonies are dominated by a single symbiont species (that is, >99% of the symbiont cells in host tissue belong to a single species). In the Caribbean and Eastern Pacific, where most high-resolution assessments have been performed, individual colonies are dominated not only by one species, but by one strain within that species. An example would be the *Acropora palmata–Symbiodinium “fitti”* association, where pairings of single host and symbiont genotypes produce holobionts that may each exhibit unique extended phenotypes (**Figure 1**; Baums et al., 2014, Parkinson et al., submitted). In fact, in studies where microsatellite markers have been used to characterize both partners, the host:symbiont genotype ratio is one:one in >70% of colonies (Goulet and Coffroth, 2003a,b; Santos et al., 2003b; Kirk et al., 2005; Pettay and LaJeunesse, 2007, 2009, 2013; Thornhill et al., 2009, 2013; Andras et al., 2011; Pettay et al., 2011; Pinzon et al., 2011; Baums et al., 2014; Prada et al., 2014b). This outcome falls in line with the predictions of basic population theory, as closely related organisms generally compete for similar resources, leading to competitive exclusion among similar species (Gause, 1934; Hardin, 1960). However, there are certainly other associations where strains from multiple *Symbiodinium* species codominate in one host colony (e.g., Rowan et al., 1997; van Oppen et al., 2001), such that the holobiont can be viewed as a more complex community. The presence of low-abundance or “background” symbionts representing <0.1% of the symbiont population may also shape some holobiont dynamics (see **Box 1**). This range of partnership complexity provides exciting potential for deconstructing the processes shaping the evolution of mutualisms across reef habitats.

**Box 1. Low abundance *Symbiodinium*.**

Given that DNA evidence is the primary means by which *Symbiodinium* are both detected and identified, our ability to quantify symbiont diversity is restricted by the molecular techniques used. Not all techniques and markers have equal resolving power (Sampayo et al., 2009). One of the most common markers, the internal transcribed spacer 2 (ITS2) of the ribosomal array, is multicopy and undergoes concerted evolution, maintaining functional and non-functional rare variants in the species population (Dover, 1982). Much debate has focused on the information lost when using denaturing gradient gel electrophoresis (DGGE) to screen out rare intragenomic variants (Apprill and Gates, 2007; Thornhill et al., 2007). This methodology conservatively underestimates total symbiont diversity within a coral colony while revealing the dominant or codominant taxa (i.e., the most numerically abundant and presumably ecologically relevant species). In the process, minor strains that comprise <5% of the total symbiont population within host tissues go unrecognized. With the development of several sensitive qPCR assays (Ulstrup and Van Oppen, 2003; Ulstrup et al., 2007; Correa et al., 2009; Mieog et al., 2009) and the advent of next generation sequencing (Kenkel et al., 2013; Green et al., 2014), it has been possible to survey the diversity of “background” populations of *Symbiodinium* below the detections limits of DGGE and traditional PCR.

In a recent survey of 26 coral taxa previously thought to be “specific” (restricted to associations with one *Symbiodinium* clade), background symbionts from multiple clades could be detected with qPCR assays in nearly all host species (Silverstein et al., 2012). When a non-symbiotic coral species was screened as a control, the assays returned false positives from putatively contaminant symbionts trapped in the mucus or gut cavity 9% of the time. This rate of natural contamination is quite high, but nevertheless, background strains are more common than previously thought. It is understood that most corals that acquire their symbionts from the environment each generation are promiscuous during early ontogeny, associating with multiple symbiont taxa that are not dominant in adults (Coffroth et al., 2001, 2006; Santos et al., 2003a; Little et al., 2004; Abrego et al., 2009a; Byler et al., 2013; Cumbo et al., 2013; Poland et al., 2013; Yamashita et al., 2013). Since the capacity for non-specific associations is present in juveniles, it is not necessarily surprising that multiple clades were detected in low abundance in adult corals (Santos et al., 2004; Baird et al., 2007; Baker and Romanski, 2007). It is currently unclear whether the presence of a background symbiont implies that it is functionally relevant to the holobiont. Though corals may have always been open to infiltration by background symbionts, host-symbiont specificities have evolved multiple times regardless.

Detection of low-abundance *Symbiodinium* cells in corals suggest that hosts may be open environments where small numbers of heterologous symbionts are entering and exiting the system on a regular basis. If commensal, these symbionts may move passively through the system without engaging in symbiosis. If parasitic, they may trigger a host rejection response or may be competitively displaced by the dominant symbiont, such that only a small number are present in a coral at a given time. Finally, if mutualistic, they may be fully engaged in the fitness of the holobiont despite their rarity. For example, rare symbionts may be important if they contribute a different but essential metabolic resource than the dominant symbiont strain (analogous to rare members of the bacterial biosphere; reviewed by Pedros-Alio, 2012), or if they can increase sufficiently in number to replace a compromised dominant symbiont should environmental conditions change (Buddemeier and Fautin, 1993; Baker et al., 2004; Berkelmans and van Oppen, 2006).

Studies are needed to distinguish between these competing scenarios. So far, the few experiments that have successfully tracked background symbionts during natural environmental extremes suggest that they are not viable sources of persistent acclimation to stress, at least in terms of replacing the dominant symbiont. After a cold-water bleaching event in the Gulf of Mexico, most *Pocillopora damicornis* colonies with mixed symbiont communities did not “shuﬄe” (c.f., Baker, 2003) to the more thermally tolerant species (McGinley et al., 2012), instead remaining stable despite environmental variability. In corals sampled before, during, and after a 2005 bleaching event in Barbados, background populations of the thermally tolerant *Symbiodinium trenchii* increased in prevalence prior to bleaching, but declined to pre-stress levels over the next 2 years of non-stressful conditions (LaJeunesse et al., 2009). However, functional relevance may not be tied directly to cell numbers (a rare strain may always be rare and yet essential). Such a hypothesis has yet to be tested in corals, though bacterial analogs are known. For example, a single rare bacterium representing 0.006% of the total cell count in peat accounted for a much larger proportion of the biome’s sulfate reduction relative to its abundance (Pester et al., 2010). This is an active research area, and despite our current data deficiency, future studies may provide more convincing evidence of the functional relevance of background *Symbiodinium*.

## INTRASPECIFIC FUNCTIONAL DIVERSITY IN CORALS: CLASSIC STUDIES

Traditionally, common garden and reciprocal transplant experiments have been used to test for functional differences of genotypes in plants (e.g., Hufford and Mazer, 2003) and corals (Potts, 1984; Edmunds, 1994; Bruno and Edmunds, 1998; D’Croz and Mate, 2004; Smith et al., 2007). Typically, colonies from environmentally distinct sites (e.g., shallow vs. deep or inshore vs. offshore) are reciprocally transplanted to test how they perform relative to native corals. In parallel, colonies from both sites may be transplanted to a third location to test how they perform relative to each other in a new common environment. As one might expect, studies on reef-building corals have found species that are characterized by generalist genotypes (Smith et al., 2007), species that show local adaptation (D’Croz and Mate, 2004; Kenkel et al., 2013), and species that harbor both generalist and specialist genotypes (Potts, 1984). Such studies address the performance of the specific combination of coral and *Symbiodinium* genotypes in the experimental units. However, the relative contribution of each partner to holobiont performance has been difficult to measure.

Prior to the mid-1990s, confirmation of the distinctness or clonality of coral colonies was difficult because of the lack of genetic data and the fact that coral clones are generally impossible to distinguish visually (even histo-incompatibility proved unreliable; Heyward and Stoddart, 1985). For example, in a classic common garden reciprocal transplant experiment, Potts (1984) mounted clonal fragments of *Acropora* sp. sourced from each of five environments from a single reef onto common wire grids. Five replicate grids were distributed among the five locations. Source location (a proxy for host genet) drove non-random differences in growth rate and survivorship among individual colonies in shared environments. After eight years of observation, colonies with different origins did not converge on a common morphology to match the native colonies at their new locations, indicating low phenotypic plasticity in this coral (at least morphologically) and further supporting a genetic component of coral performance. However, the corals sampled for this study may have included two cryptic species that in some environments can only be distinguished with molecular techniques (Potts, 1984; Ayre et al., 1991).

In another example, host genotype effects on thermotolerance were examined (Edmunds, 1994). To minimize the chance of incorrectly assigning genets, patches of *Orbicella ( = Montastraea) annularis* complex that were physically clustered in groups attached by contiguous skeleton but unconnected by coral tissue were considered as clones of the same genotype because such a formation suggests a common origin. The author showed that bleaching colonies were aggregated rather than randomly distributed on the reef, and that these aggregations corresponded to genotype identities. While the spatial distribution of bleaching colonies might alternatively be explained by the distribution of colonies with distinct *Symbiodinium* associations and therefore thermotolerances, it is unlikely that the experimental colonies harbored different symbiont species. This is because the corals were located at a common depth over a small spatial scale, reducing the number of light microhabitats that lead to unique symbiont associations within the host species complex (Rowan et al., 1997). In a second experiment, subfragments from large colonies of *Porites porites* located more than 15 m apart (thus suggesting they belonged to different genets) were experimentally exposed to elevated temperatures for three days and their symbiont densities were measured. Despite having similar densities at the start of the experiment, the putatively distinct genotypes showed different rates of symbiont loss (or, in one case, gain) after thermal stress exposure (Edmunds, 1994).

The coral literature is rife with similar examples where genotype level effects seemed apparent, but actual genotypes were not resolved explicitly. Given that the spatial range over which host ramets of the same genet have been distributed (e.g., from <1 to >70 m in *Acropora palmata*; Baums et al., 2006), it may not be appropriate to assume that by swimming a certain distance, the chance of collecting a clonal colony is greatly reduced. For fine-scale ecological questions, it will be necessary to incorporate molecular confirmation of intraspecific diversity. As genomics-empowered tools become less expensive and more accessible, a greater number of studies are taking advantage of fine-scale resolution.

## INTRASPECIFIC FUNCTIONAL DIVERSITY IN CORALS: GENOMICS-EMPOWERED STUDIES

A series of recent work on the Mediterranean Red Coral (*Corallium rubrum*) demonstrates the utility of a genomics approach to studies of marine evolutionary ecology. This particular coral lacks *Symbiodinium*, reducing the complexity of the system. First, neutral microsatellite markers were used to differentiate populations of *C. rubrum* (Ledoux et al., 2010a,b; Costantini et al., 2011). Populations were structured along a depth gradient that reflected distinct, stable thermal environments. This genetic structure corresponded with variability in *C. rubrum* thermal stress limits (Torrents et al., 2008). Since the multilocus genotypes of each colony were established, individuals from each population could be targeted to assess physiology. Colonies were subfragmented and exposed to various heat stress regimes in common garden aquaria, while the expression of key heat shock proteins were monitored via qPCR (Haguenauer et al., 2013). After assessing variability in gene expression among individuals within different populations, the authors found evidence consistent with local adaptation driven by environmental variability, and argued for a trade-off between reduced responsiveness of metabolic genes and frontloading of thermotolerance genes. Critically, environmental heterogeneity at shallow sites seemed to select for phenotypically plastic individuals, as reflected by high genetic variability in the shallow population versus low genetic variability in the populations at depth. This work emphasizes the potential importance of cryptic diversity in coral communities and the significance of marginal populations in providing evolutionary novelty (Bell and Gonzalez, 2011; Boulay et al., 2014). It also exemplifies a useful strategy for investigating genotype level effects driving thermal adaptation in symbiotic corals.

The reductive approach of assessing the performance of either the host or symbiont in isolation is more difficult for symbiotic scleractinian corals. One methodology is to experiment with coral larvae, which often lack *Symbiodinium* prior to settlement. Crosses of gametes collected from distinct adult genets produce large batches of offspring with known heritage. Controlled crosses between adjacent *Acropora palmata* individuals showed that full sibling larval batches were unequally affected by thermal stress, which influenced swimming speeds and developmental rates (Baums et al., 2013). The same larval batches exhibited diverse transcriptional responses to thermal stress depending on their heritage (Polato et al., 2013), revealing a higher-than-expected degree of molecular variation in this endangered coral species. Among *Acropora palmata* adults, some individuals were sexually incompatible (Baums et al., 2013). This was not due to general infertility as most individuals were capable of producing viable larvae when crossed with a compatible genotype. Clearly, intraspecific diversity has fitness consequences in corals. In another experiment, Polato et al. (2010) identified colonies of *Orbicella faveolata* at two distant locations that belonged to one panmictic population according to neutral markers. At each location, locally derived aposymbiotic larval batches were exposed to a common thermal stress. The larvae exhibited both shared and location-specific transcriptional responses, strongly suggesting the existence of local adaptation despite ongoing gene flow among locations.

Because some *Symbiodinium* can be maintained in culture, their performance can be measured independent of a host. *Symbiodinium goreaui* is a host-generalist symbiont featuring a global distribution (LaJeunesse, 2005). In one study, *Symbiodinium goreaui* was identified in two *Acropora tenuis* reefs located several hundred kilometers apart with average temperatures differences of ~2°C (Howells et al., 2009). After establishing via microsatellite genotyping that these reefs are likely inhabited by distinct populations of *Symbiodinium goreaui*, symbionts from each population were isolated and cultured (Howells et al., 2012). Cultures were then exposed to elevated temperatures, and photochemical performance was monitored. *Symbiodinium goreaui* cultured from the warmer reef population showed a smaller decline in photochemical performance at elevated temperature relative to the population from the cooler reef, even after >30 asexual generations in culture. Similar *in vitro* experiments have shown within-species differences in physiology (see *Symbiodinium* Growth Rates in Culture). Thus, when separated, both corals and *Symbiodinium* show intraspecific variation in thermotolerance that appears to have a heritable genetic component—the raw material of natural selection.

Howells et al. (2012) further tested whether intraspecific variation influences holobiont performance when the host and symbiont are combined. They used the distinct *Symbiodinium goreaui* populations to inoculate aposymbiotic larvae of the coral *Acropora millepora*. After growing to a sufficient size, symbiotic coral juveniles were then exposed to ambient or elevated temperatures, and both symbiont and host physiology were assessed. The symbiont population from the warmer reef showed optimal photochemical performance at elevated temperature, and coral juveniles associating with these symbionts grew rapidly with no signs of bleaching and minimal mortality at high temperature. In contrast, the symbiont population from the cooler reef experienced chronic photodamage at high temperature, and the juveniles inoculated with this population grew slowly and suffered high bleaching and mortality at high temperature. Symbiont and host thermotolerance correlated, showing a strong influence of symbiont physiology on holobiont performance even below the species level. In a similar vein, Kenkel et al. (2013) used microsatellites and identified performance differences among two populations of the coral *Porites astreoides*. In this case, both hosted the same *Symbiodinium* species as determined by characterization of the symbiont community using high-throughput sequencing of the ITS2 marker. Host structure appeared to be maintained by differences in variable inshore vs. stable offshore thermal regimes. In a common garden, offshore holobionts were less tolerant of experimental heat stress, showing elevated bleaching and reduced growth compared to inshore holobionts. Despite the homogeneity of the symbiont population, *Symbiodinium* in offshore hosts experienced lower photochemical efficiency during heat stress than those associating with inshore hosts. These results support the contention that the host plays an important role in holobiont thermotolerance (Baird et al., 2009a). Moreover, it is not just the host species, but intraspecific populations that may determine performance.

To assess host and symbiont adaptive potential, Csaszar et al. (2010) identified two coral populations of a single species (*Acropora millepora*). Each population associated with a different symbiont species. Heritability estimates for key thermal response traits within each host population showed the symbionts to be relatively more capable of adapting to climate change than the host. However, as the authors recognized, while hosts were genotyped to the level of individuals, symbionts were only resolved to the sub-cladal type (approximately species) level. Though the relative comparisons between host and symbiont heritability must be interpreted with caution, this study sets an excellent precedent, as it is one of the few to both measure intraspecific trait variation in coral hosts and confirm the unique identity of the host genets involved.

## PRELIMINARY EVIDENCE IN A GENOMICS AGE

While the previously mentioned studies mostly examined intraspecific variation at the population level, genotype level effects have only rarely been explored (Baums et al., 2013; Polato et al., 2013). Now that both major components of the coral holobiont can be genotyped to individuals, the doors have opened for high-resolution investigations of partner interactions. Here we highlight preliminary evidence that variation at the genotype-level may be extensive in both corals and *Symbiodinium*, and that unique partner pairings drive unique responses to stress. This work tests the first of our major hypotheses; that interactions between partners contribute to functional diversity that may subsequently be acted upon by selection. We argue that to truly understand how corals may respond to the myriad selective pressures of a changing climate it will be necessary to assess the contribution of intraspecific diversity to holobiont performance.

### CORAL GROWTH IN RESTORATION NURSERIES

With global reef degradation reaching alarming levels, marine managers have developed methods to rear coral fragments *in situ* for restoration purposes. A typical “coral gardening” approach involves several steps: donor colonies are identified and fragmented; the pieces are attached to artificial substrate; the fragments are grown together in a common nursery plot; ultimately, these aquacultured colonies are outplanted to depauperate reefs (Rinkevich, 1995, 2005). The goal is to increase coral biomass, diversity, and reproductive capacity, as well as to restore the reef ecosystem and associated fauna (Precht, 2006). During the growth phase, the underwater nurseries serve as common gardens where environmental conditions are roughly equivalent for all colonies, and observed differences can be attributed mostly to genetic effects (Baums, 2008). Maternal effects or acclimation to the donor colony’s source environment can carry over to affect performance in the nursery, but these factors have been difficult to assess. Restoration nurseries have greatly expanded in the Caribbean, where the endangered *Acropora cervicornis* and *Acropora palmata* have been targeted for extensive management (Lirman et al., 2010; Johnson et al., 2011; Young et al., 2012). As part of the process, hundreds of colonies in the Florida Reef Tract have been genotyped at multilocus microsatellite markers (e.g., Baums et al., 2010), and many have been monitored for growth and mortality for several years (Griffin et al., 2012; Lirman et al., submitted).

These nurseries provide a unique and under-utilized resource for investigations of genetic influence on coral performance. The few studies that have been conducted with nursery-reared colonies all point to intraspecific genotype effects on growth. For example, Bowden-Kerby (2008) reared genets of acroporid corals from both forereef and backreef environments in a common garden backreef nursery. In contrast to the study of Potts (1984), here source population (a proxy for host/symbiont genotype) was more important than environment in determining growth rate; source was determined to be a significant factor in 75% of tests compared to 44% for environment. Forrester et al. (2013) transplanted *Acropora palmata* fragments from two source locations to a common garden at a third. In the first year, there were no observed differences between groups, but when the experiment was repeated, growth rate varied by source. In a concurrent experiment, colonies were subdivided into fragments and reciprocally transplanted to “home” and “away” environments. Clonal fragments moved “away” grew more slowly, revealing a slight home-field advantage and a combined influence of both environment and genotype.

**FIGURE 2:**
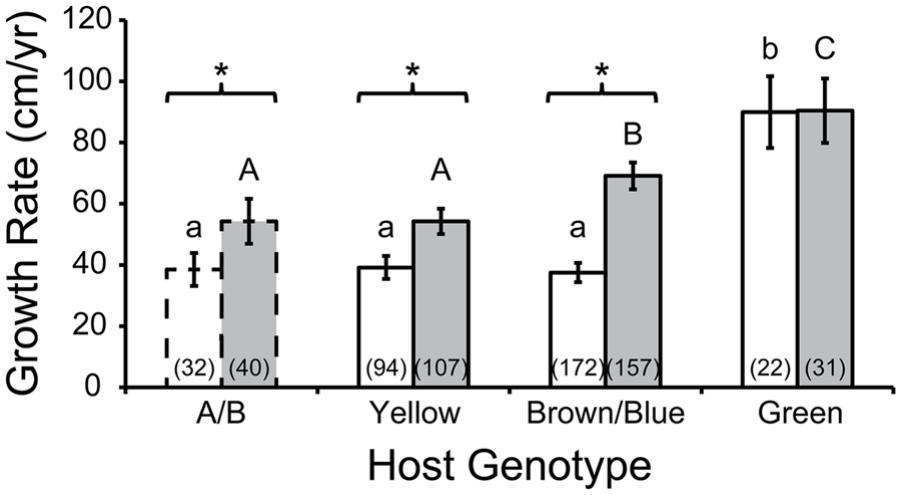
***Acropora cervicornis* colony growth as a function of host genotype.** The Green host genotype had higher growth rates (cm/year) then genotypes A/B, Yellow, and Brown/Blue in shallow water (white bars; similar growth rates indicated by common lower case letters, Tukey’s HSD; *p* < 0.05) and in deep water (gray bars, similar growth rates indicated by common upper case letters, Tukey’s HSD; *p* < 0.05). Growth rates were usually higher in deep compared to shallow colonies of a given genotype (asterisks: *t*-test; *p* < 0.05). Numbers in parentheses indicate sample size (number of colonies). Error bars depict 95% confidence intervals. Host A/B is bordered by dashed lines to emphasize that for this particular holobiont, the corresponding *Symbiodinium “fitti”* strain was distinct from the strain that was common to the other three holobionts. Data reanalyzed from Griffin et al. (2012).

Griffin et al. (2012) reared fragments of several *Acropora cervicornis* genotypes at a line nursery in Puerto Rico and confirmed the hypothesis that linear tissue extension rate varied among individuals. A re-analysis of this data set is presented here (**Figure 2**). In addition to discriminating growth rates by host genotype, we also separated colonies into depth classes by their relatively shallow (9–10.5 m) or deep (10.5–13 m) positions in the line nursery, as depth was a significant factor in model analysis (Griffin et al., 2012). We removed measurements from individuals attached to the lines by cable ties, as this method was shown to negatively affect growth (Griffin et al., 2012). To use the terminology of that study, host genotypes are referred to by color names or capital letters. Repeat genotyping of host samples derived from the nursery (rather than the donor colony, as in the original study) revealed that genotypes “A” and “B” were actually identical, as were “Blue” and “Brown,” so their measurements were pooled. Additional genotyping of the dominant symbiont associated with each colony revealed that three of the four hosts shared a clonal *Symbiodinium “fitti”* (ITS2 type A3) strain; host “A/B” associated with a unique *Symbiodinium “fitti”* strain. The “Green” host genotype grew faster than all others, regardless of depth. Identical individuals generally grew faster at greater depth. Interestingly, the “Blue/Brown” genotype deviated significantly from the “A/B” and “Yellow” genotypes when reared in deep but not shallow depths. This indicates an interaction between host genotype and environment. Symbiont genotype did not appear to affect growth, since the most deviant host genotypes shared a clonal symbiont, while two of the hosts that did not differ in growth rate at either depth associated with distinct symbionts. To test this particular hypothesis rigorously, it will be necessary to track the growth rates of ramets of the same host genet each associating with distinct symbiont genotypes; such cases are difficult (though not impossible) to find in nature (Baums et al., 2014).

### *Symbiodinium* GROWTH RATES IN CULTURE

It has long been possible to culture *Symbiodinium* independent of the host in artificial media (McLaughlin and Zahl, 1959). By now a great many studies have been performed *in vitro*, revealing key physiological differences among *Symbiodinium* in terms of cold tolerance (Thornhill et al., 2008a; McBride et al., 2009), heat tolerance (Robison and Warner, 2006; Suggett et al., 2008), light tolerance (Iglesias-Prieto and Trench, 1994, 1997a; Hennige et al., 2009), and acidification tolerance (Brading et al., 2011). Typical phenotypic traits that have been monitored under different environmental conditions include culture growth rates and photochemical efficiencies (e.g., Robison and Warner, 2006; Thornhill et al., 2008a). Given the state of *Symbiodinium* taxonomy prior to the 1990s, most early work assumed the physiology of a few cultures was representative of the entire genus. Over the years, more studies have incorporated clades, types, and species designations, broadening our understanding of the extensive physiological diversity within *Symbiodinium*, but none have resolved individuals within species.

Using a hierarchical molecular approach, two species of Clade B *Symbiodinium* were recently delineated with a combination of nuclear, mitochondrial, and chloroplast markers (LaJeunesse et al., 2012). *Symbiodinium minutum* associates with the globally distributed anemone *Aiptasia* sp. in tropical waters, while *Symbiodinium psygmophilum*, despite being present in the tropics, is cold-tolerant and typically engages in symbiosis with the scleractinian corals *Astrangia poculata, Cladocora caespitosa,* and *Oculina patogonica* in high latitudes of the Atlantic Ocean. In a preliminary experiment designed to test the hypothesis that phenotypic differences could be detected among genotypes within and between *Symbiodinium* species, we reared several monoclonal cultures of *Symbiodinium minutum* and *Symbiodinium psygmophilum* genotypes under identical temperature and light regimes and monitored growth rates (in terms of asexual propagation of cells). We used the micro-culture methods of Rogers and Davis (2006) as a guide, and reared all cultures in ASP-8A media (Ahles, 1967). First, genotype uniqueness was confirmed with microsatellite repeat length variation (i.e., different alleles) at nuclear marker *Sym15* (Pettay and LaJeunesse, 2007) and sequence variation at chloroplast *psbA^ncr^* (Moore et al., 2003; LaJeunesse and Thornhill, 2011) for each culture of each species. Next, individual cells from synchronized cultures (*n* = 3 genotypes per species) were transferred to 96-well plates via cell sorter such that each culture was represented in sixteen replicate wells with ~5 cells each at the start of the experiment. Plates were incubated at 25°C and a 12:12 light/dark photoperiod at 60 microeinsteins. As cells divided asexually, plates were observed under a microscope at 400X magnification and total cell counts were recorded at noon every 2 days for 2 weeks. The growth rates were exponential, so data were log transformed and fit to a linear regression. The slope of the line was recorded as the growth metric per replicate well. The entire experiment was repeated twice.

The *Symbiodinium psygmophilum* culture PurpPFlex failed to grow (as occasionally happens with recent transfers of older cultures, such as in this case), so ultimately we collected data from three *Symbiodinium minutum* genotypes (Mf1.05b, rt-002, and rt-351) and two *Symbiodinium psygmophilum* genotypes (Mf10.14b.02 and rt-141). Initial growth was highly variable until at least ten cells were present in each well, and cell counts became difficult after concentrations reached >200 cells/well, so we only included in our analysis wells with time series data between this count range. After failing to detect differences between experiments (*t*-test, *t*_(101)_ = 1.25, *p* = 0.216), data from each run were combined and analyzed together.

**FIGURE 3:**
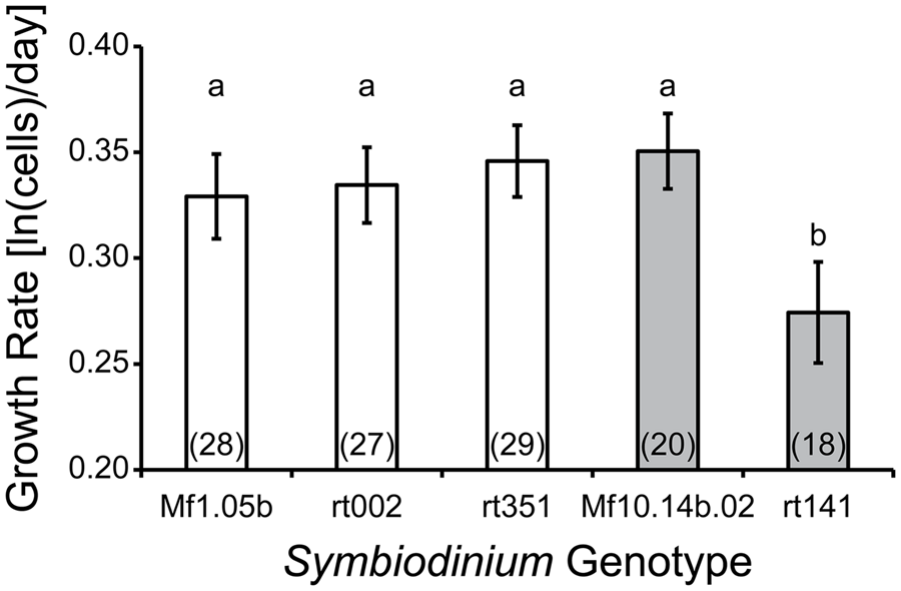
**Symbiodinium culture growth as a function of genotype.** *S. minutum* genotypes (white bars) showed little variation in growth rates compared to *S. psygmophilum* genotypes (gray bars). Letters indicate statistically different growth rate groupings (Tukey’s HSD; *p* < 0.05). Numbers in parentheses indicate sample size (number of wells). Error bars depict 95% confidence intervals. Denecke et al. (unpublished data).

We noted a difference in average growth rate between species, reported here as ln(cells/day) ± 95% Confidence Interval. For *Symbiodinium minutum,* the growth rate was 0.34 ± 0.01, while for *Symbiodinium psygmophilum* it was 0.31 ± 0.02 (ANOVA, *F*_(1,120)_ = 4.97, *p* = 0.028). When separated by genotype, it became clear this effect was driven by the *Symbiodinium psygmophilum* culture rt-141, which had much lower growth rates than all other cultures regardless of species (ANOVA, *F*_(4,117)_ = 7.39, *p* < 0.001; **Figure 3**). The diversity in growth rates among *Symbiodinium psygmophilum* may reflect the genetic diversity within this species, which exceeds that of *Symbiodinium minutum* (LaJeunesse et al., 2012). The key result is that phenotypic variation among genotypes within *Symbiodinium* species can potentially exceed that found between members of different species. This situation is not uncommon in nature (Bangert et al., 2006), but to date, the concept of intraspecific variation within *Symbiodinium* species has largely been ignored. A vast preponderance of reef ecology studies only measure symbiont phenotypes at the low-resolution “clade” or intermediate-resolution “type” level. Using crude averages from these higher-order taxonomic rankings may miss important dynamics taking place among or within species. Further experimentation with more *Symbiodinium* genotypes (both *in vitro* and *in hospite*) will be necessary to confirm these findings. The fact that such patterns can be found even among a small number of strains implies that, much like in corals, intraspecific variation in symbiont physiology may be extensive.

### HOST GENOTYPE EFFECTS ON CLONAL SYMBIONT PERFORMANCE

In their analysis of host and symbiont population interactions, Howells et al. (2012) showed that intraspecific variation among *Symbiodinium* influenced the growth of host juveniles in a laboratory setting. But does intraspecific variation among hosts influence symbiont performance? To address this question, we recently took advantage of the *Acropora palmata–Symbiodinium “fitti”* association, wherein individual host colonies usually associate with only one clonal symbiont strain (Baums et al., 2014). Distinct coral genets that shared a clonal *Symbiodinium “fitti”* strain were identified growing close to each other within a natural common garden. Highly sensitive qPCR assays established that no other *Symbiodinium* could be detected within the colonies. Fragments were removed, exposed to cold shock *ex situ* (10°C for 3 days), and monitored for photochemical efficiency changes and acute host transcriptional responses. We found that the photochemical response of the symbiont strain varied depending on which host genotype it associated with (Parkinson et al., submitted). Because all measured *Symbiodinium* were clonal and environmental variation was reduced by the proximity of the colonies, the most parsimonious explanation was that physiological variation among host genotypes drove photochemical differences among the clonal symbiont strains. Experiments designed to test for intraspecific variation should make sure that individual histories are not a confounding factor; the natural common garden proved advantageous for that purpose here.

In a subset of the holobionts exposed to cold, symbiont photochemical efficiency was phenotypically buffered (Waddington, 1942; Bradshaw, 1965; Reusch, 2014), meaning the reaction norm changed relatively little with environmental perturbation. In other host backgrounds, the symbiont strain’s response was less buffered. Host expression of iron sequestering and oxygen stress signaling genes correlated with these differences in symbiont performance, suggesting that variation in iron microhabitat and/or redox sensitivity among hosts may mediate clonal symbiont performance during stress. Anecdotally (because sample size was small), the colonies that participated in the annual spawning event had the most buffered symbiont responses. Those colonies with less buffered symbiont responses did not spawn. This result suggests a possible fitness consequence of genotype interactions among holobionts, highlighting the potential evolutionary importance of intraspecific diversity among coral mutualists.

### METABOLOMIC ANALYSIS OF SYMBIOTIC AND NON-SYMBIOTIC POLYPS

The *Astrangia poculata–Symbiodinium psygmophilum* association has been proposed as a model system for investigating coral–algal symbiosis. This scleractinian hard coral is more amenable to aquaculture than exclusively tropical species and exists across a broad latitudinal and temperature range. Uniquely, *Astrangia poculata* colonies often feature both symbiotic and non-symbiotic polyps within the same colony under non-stressful conditions. This attribute allows for experimental investigation into the molecular features that mediate successful symbiotic interactions among hosts and symbionts while controlling for partner genotypes. We generated metabolomic profiles for symbiotic and non-symbiotic polyps dissected from each of three *Astrangia poculata* colonies to provide another example of the insights that can be gained when intraspecific diversity is accounted for in the experimental designs. We also analyzed a *Symbiodinium psygmophilum* monoclonal culture (isolated from a tentacle of *Astrangia poculata*). Methods generally followed Gordon et al. (2013) with minor modifications. Target tissues were snap frozen in liquid nitrogen within 1 min of sampling, then metabolites were extracted in isopropanol:acetonitrile:water (3:3:2) solution. The samples were separated on a Shimadzu 20R UFLC high-performance liquid chromatography system using a C_18_ column. Mass spectra and tandem mass spectra were obtained in both positive and negative ion mode on an AB SCIEX 5600 Triple TOF. The resulting LC-MS profiles were Pareto transformed to reduce bias from metabolites with large fold changes while preserving the rank and dimensionality of the data (van den Berg et al., 2006).

**FIGURE 4:**
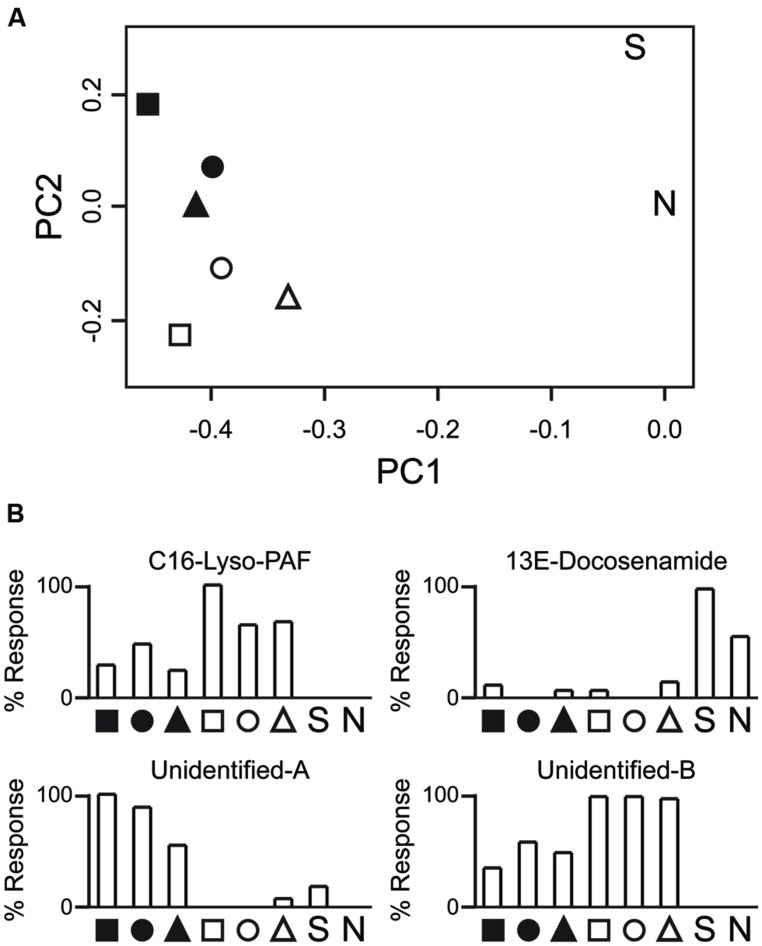
**Preliminary analysis of the *Astrangia poculata–Symbiodinium psygmophilum* metabolome. (A)** Principle component analysis of metabolite profiles. Shown are principle components 1 and 2 (x- and y-axis, respectively) of Pareto-transformed metabolite data. Shapes indicate host genotype (*n* = 3). Black fills correspond to symbiont-rich polyps. White fills correspond to nearly symbiont-free polyps. “S” indicates a sample of a *Symbiodinium psygmophilum* monoculture. “N” indicates a negative control (purified water). *Astrangia poculata* samples cluster by the symbiotic state of the polyps rather than by host genotype. **(B)** Repre sentative profiles for specific metabolites. C16-Lyso-PAF was abundant in non-symbiotic polyps but low in symbiotic polyps and absent in *Symbiodinium* culture. 13E-Docosenamide was mainly present in *Symbiodinium* culture but not in coral tissue. The two unidentified compounds are characteristic of metabolites with greater detection in symbiotic (Unidentified-A) or non-symbiotic (Unidentified-B) polyps. Polato et al. (unpublished data).

Principle component analysis (PCA) clustered polyps by symbiont state more strongly than host genotype (**Figure 4A**). PCA loadings revealed ~4000 compounds (including isotopic and monoisotopic peaks) that were mainly present in only one of the symbiotic states, driving group clustering. For example, a platelet activating factor (PAF) was observed at much higher levels in non-symbiotic polyps (**Figure 4B**). This metabolite has multiple functions in humans, and may play a role in intracellular signaling (Venable et al., 1993). The single *Symbiodinium* sample fell far from either of the holobiont clusters in the PCA. Certain compounds were observed only in the *Symbiodinium* sample, such as 13E-Docosenamide, the function of which is unclear in *Symbiodinium* (it has been found in the cerebrospinal fluid of mammals; Cravatt et al., 1995). Unfortunately, a majority of metabolites could not be easily annotated, and further work will be required to characterize them. Controlled contrasts should reveal key players in the metabolic interactions that allow the symbiosis to persist. Being able to compare fragments of the same host genotype in two symbiotic states reduces the problem of working with non-model coral species that contain a large amount of genetic variation. That variation would otherwise obscure patterns. This is but one example of how new technologies, when applied to combined and isolated components of the holobiont, will facilitate new insights into marine endosymbiotic mutualisms.

## COEVOLUTIONARY CONTEXT AND CLIMATE CHANGE

Mutualisms in general (Kiers et al., 2010) and coral–algal associations in particular (Hoegh-Guldberg et al., 2007) are threatened by a changing climate and anthropogenic disturbance. Aside from the extreme case of mutual extinction (Dunn et al., 2009), other negative evolutionary outcomes of changing environmental conditions may include shifts from mutualism to antagonism, switches to inferior partners, and mutualism abandonment (Kiers et al., 2010). Unequal responses to climate shifts between partners can contribute to mutualism breakdown (Warren and Bradford, 2014). Such breakdown is apparent in coral systems, where the “coral bleaching” phenomenon (when hosts and symbionts dissociate due to stress) takes place at temperatures below the upper thermal limits of most free-living microalgae (Berry and Bjorkman, 1980). There is a unique aspect to engaging in symbiosis that makes the intact association more sensitive to temperature changes; this is likely due to the consequences of an oxygen-sensitive animal taking on a photosynthetic symbiont that generates reactive oxygen species under elevated light and temperature conditions (Lesser, 2006; Baird et al., 2009a). While many efforts have been made to assess the adaptive potential of coral holobionts facing rising sea surface temperatures, almost none have considered intraspecific trait variation (but see Csaszar et al., 2010). Such investigation will be needed to more accurately predict the role of coevolution in the coral holobiont response to climate change.

Many corals transmit their symbionts vertically by provisioning eggs with *Symbiodinium* cells (Hirose et al., 2008), but most spawn symbiont-free gametes or larvae (Baird et al., 2009b), and therefore must acquire their algal complement from the environment. In a closed vertical system it is easier to accept that tight coevolution takes place; it is less clear how coevolution plays out when partner genomes are uncoupled every host generation. And yet, there is remarkable stability among holobionts with horizontal transmission. The Caribbean broadcasters in the *Orbicella* genus appear flexible at the clade level (associating with members of Clades A, B, C, and D), but are quite specific at finer-scale resolution, hosting only a few species within each clade (Thornhill et al., 2014). The two lineages of the Caribbean gorgonian *Eunicea flexuosa* each associate exclusively with a corresponding Clade B symbiont (Prada et al., 2014b), while the Caribbean scleractinian *Acropora palmata* typically associates with *Symbiodinium “fitti”* (Baums et al., 2014). These examples, along with a number of other studies and data sets, clearly demonstrate that coevolution takes place in coral–algal systems, with unique host and symbiont combinations (holobionts) being the units of selection (Iglesias-Prieto and Trench, 1997b; LaJeunesse et al., 2004, 2010; LaJeunesse, 2005; Reshef et al., 2006; Finney et al., 2010; Correa and Baker, 2011; Lesser et al., 2013; Thornhill et al., 2013, 2014; Prada et al., 2014b).

We can view the holobiont as a unit of selection because survival may depend on a given host and symbiont genotype combination. It is less clear whether holobionts can be considered strict units of evolution (Maynard-Smith, 1991; Frank, 2011; Heath and Stinchcombe, 2014). Coevolution of the holobiont as a unit does not necessarily follow directly from selection on its components. The host and symbiont are organisms with their own evolutionary paths; the frequent uncoupling of host and symbiont genomes prevents direct co-heritability of genetic information (Maynard-Smith, 1991). However, this does not prevent the species from coevolving, since specialized associations clearly exist (LaJeunesse, 2002). Coevolution despite vertical *Symbiodinium* transmission can be explained by the processes of ecological selection via host-specialization (Thornhill et al., 2014), with or without geographic isolation (Flaxman et al., 2014). Divergent selection should act on intraspecific variation to favor adaptations that increase *Symbiodinium* fitness in a given host intracellular habitat, removing suboptimal generalist genotypes. The *Eunicea* association provides a good example where both host and symbiont lineages are relatively recently diverged and the *Symbiodinium* are host-specialized (Prada et al., 2014b).

Aspects of population biology that may shed light on coevolutionary capacity are patterns of population genetic structure and gene flow. Based on the current evidence, population genetic structure does not match between coral host and algal symbiont (Andras et al., 2011, 2013; Baums et al., 2014). Adaptation to thermal and ocean acidification stress is likely ongoing but those adaptations that require reciprocal changes in the mutualistic partners (e.g., pathways involved in exchange of nutrients) will be spread inefficiently if dispersal scale is not matched between partners. For example, in *Acropora palmata* the host is divided into two large populations encompassing the eastern and western Caribbean (Baums et al., 2005b). At the same time, the dominant symbiont (*Symbiodinium “fitti”*), consists of seven populations, each found over smaller geographic regions (Baums et al., 2014). Thus a beneficial adaptation arising in *Symbiodinium “fitti”* may only efficiently rise to high frequency in parts of the host range. However, even weak selection can be sufficient to spread advantageous alleles throughout structured populations, in part because fixation times for such alleles are greatly reduced relative to their neutral counterparts (Slatkin, 1976; Rieseberg et al., 2004). Patterns of gene flow can vary substantially among coral hosts from small to large geographic scales (reviewed by Baums, 2008). We expect the same to be true for *Symbiodinium* species. Hence, additional studies are needed that resolve the population genetic structure of both partners simultaneously.

Little theoretical work has been done to understand how population genetic structure should be matched between hosts and symbionts. Work on parasites suggests that population structure should be smaller scale in the parasite compared to the host population (as found by Dybdahl and Lively, 1996), though there are examples of the opposite case (Martinez et al., 1999) and balanced structure (Mulvey et al., 1991). However, the traditional Red Queen model of rapid antagonistic coevolution does not seem appropriate for mutualisms, where fitness consequences of interactions are measured in gains rather than losses. An alternative model for mutualisms based on game theory, the Red King hypothesis (Bergstrom and Lachmann, 2003), predicts that unbalanced evolutionary rates among partner species can be stable. Currently, this model is not spatially explicit—it cannot account for local adaptation to environmental gradients such as light, for example—but nevertheless makes interesting predictions. According to Red King, the host is assumed to be “enslaving” the faster-evolving symbiont (Hilbe et al., 2013) by repeatedly “demanding” over evolutionary time scales that more opportunistic symbiont genotypes evolve back toward being more generous. The Red King hypothesis may need to be modified to account for the one-to-many interactions between a coral colony and individual *Symbiodinium* cells (Gokhale and Traulsen, 2012). Finally, such models will require empirical data accounting for both inter- and intraspecific diversity and population structure in both partners. Results might provide important insight when predicting the effects of climate change on marine mutualisms.

## FUTURE DIRECTIONS

Consideration of intraspecific diversity in experimental designs will likely improve the predictive value of models of climate adaptation in corals. For example, when climate projections do not incorporate adaptive processes such as genetic adaptation, they predict 20–80% more mass bleaching events in a given period than when such processes are included (Logan et al., 2014). Adaptation-free models over-predict the current frequency of bleaching, which indicates that adaptive processes are likely ongoing. Indeed, rapid adaptation and acclimation to thermal stress have been demonstrated among corals exposed to highly variable temperatures (Palumbi et al., 2014). Intraspecific diversity may represent a component of adaptive capacity to increased temperature in corals (Baums, 2008; Baums et al., 2013), although rare beneficial alleles can spread rapidly even when diversity is low. We would predict a link between intraspecific diversity and bleaching resistance, much like the classic link between diversity and infectious disease resistance (O’Brien and Evermann, 1988). If an empirical link can be made, this information can be incorporated into models projecting the survival of corals.

There are several areas where the development of new techniques will provide further insight in to the nature of marine mutualisms. The difficulty of aquaculturing corals has always presented a challenge to molecular studies in this system. Rearing of an F2 generation for traditional genetic experiments has previously been intractable. Only recently has successful culturing of corals from gametes to sexual maturity taken place (Iwao et al., 2010; Baria et al., 2012). These colonies spawned after three or four years of growth, indicating that the rearing of F2 generations to sexual competence for backcrosses will require at least six years for these species. Further complications stem from the symbiotic promiscuity of larvae, which may take more than three years to reflect the algal complement of stable adult colonies (Abrego et al., 2009b). Despite these issues, new technologies are providing different avenues for molecular characterization of corals. For example, Lundgren et al. (2013) recently used next generation sequencing to characterize a suite of single nucleotide polymorphisms (SNPs) that correlate with environmental variables in populations of scleractinian corals on the Great Barrier Reef. Five SNPs for *Acropora millepora* and three SNPs for *Pocillopora damicornis* exhibited likely signatures of selection. These markers may serve as quantitative trait loci for stress tolerance, a critical tool for managers attempting to identify particularly resilient genotypes for restoration purposes.

In parallel with the development of microsatellite markers to distinguish coral and algal individuals, efforts have been made to elucidate the taxonomic diversity of coral-associated microbes, cryptic invertebrates, and more transient associates such as reef fish. An integrative approach that simultaneously assesses diversity across all these community-levels would provide a comprehensive understanding of how coral genotypic diversity affects and is affected by reef community diversity. This can be accomplished by combining surveys of natural coral stands, manipulation of *in situ* common gardens, and *ex situ* experiments. Even at small spatial scales, natural variation in genotypic evenness and richness is common within and across species, ranging from minimal clonal replication to reefs dominated by just one genet (Hunter, 1993; Ayre and Hughes, 2000; Miller and Ayre, 2004; Baums et al., 2006; Boulay et al., 2014). By tracking the functional and taxonomic diversity of associated micro- and macro-scale assemblages over time in plots of varying host and symbiont genotypic diversity or composition, it will be possible to quantify the link between diversity and community dynamics. We would predict that host and *Symbiodinium* genotypic diversity positively correlate with microbial and epifaunal community diversity. The incorporation of environmental stressors in such designs will help to assess the direct effects of those stressors as well as the indirect effects of diversity and composition on both ecosystem function and resilience, potentially informing conservation and restoration strategies (Srivastava and Vellend, 2005). Again, we would predict a positive association between holobiont genotypic diversity and resilience. These types of studies would address our second major hypothesis; that reef community dynamics are influenced by intraspecific diversity among corals.

An interesting application of fine-scale techniques will be to examine the coral colony landscape in terms of the distributions of different symbiont genotypes throughout host tissues. Do *Symbiodinium* stratify not only based on light regime (e.g., top, bottom, and sides of colonies), but also within specific host tissues (e.g., tentacles)? Can multiple symbiont species or genotypes within a species occupy a single symbiosome within a single host cell? Laser-capture microdissection (Espina et al., 2006) has already been used to isolate targeted bacterial endosymbionts of *Siboglinum fiordicum*, a tube worm (Thornhill et al., 2008b). The same technology could be applied to isolate *Symbiodinium* among non-calcifying hosts *in hospite*, and be coupled with transcriptomic or metabolomic profiling. Because somatic mutations in the undifferentiated host germ line can propagate as corals age (reviewed by Van Oppen et al., 2011), and early larval fusion can generate chimeras (Frank et al., 1997; Barki et al., 2002; Puill-Stephan et al., 2009), it will also be interesting to map host genotypic mosaicism within a colony and to see if this influences symbiont associations in any way.

Further research into the physiology and ecology of background *Symbiodinium* is required to determine the role of this diversity in coral holobionts. Manipulating background strains will be difficult. A first step would be rearing healthy, completely symbiont-free corals, much like sterile mice reared without gut bacteria. With current aquaculture techniques, this is impossible for scleractinian hard corals. Progress has been made in the model anemone *Aiptasia* sp. (Weis et al., 2008). Though they lack the biomineralization processes of hard corals, *Aiptasia* represent a promising first step for several reasons. It is easy to produce clonal replicates, novel associations with heterologous symbionts are possible, and the same individuals can be inoculated, bleached, and re-inoculated experimentally in an aquarium setting. Moreover, genomic resources are available for the host and the homologous symbiont, *Symbiodinium. minutum* (Sunagawa et al., 2009; Bayer et al., 2012; Lehnert et al., 2012; Shoguchi et al., 2013). This system may be well-suited for establishing whether background *Symbiodinium* are functionally relevant during normal and stressful conditions. Additional transcriptomic, metabolomic, and proteomic characterizations of different *Symbiodinium* are ongoing. By contrasting molecular phenotypes at both coarse resolution (e.g., between clades; Ladner et al., 2012; Barshis et al., 2014) and fine-scale resolution (e.g., between species within clades and between individuals within species), we will begin to decipher the mechanisms by which evolution gave rise to the current diversity of *Symbiodinium.*

## CONCLUSION

Intraspecific variation is a major component of terrestrial mutualisms, affecting ecological interactions between proximate symbiotic species as well as higher-order community dynamics. Our understanding of such forces in marine endosymbiotic associations is lacking. We have reviewed some of the current literature and presented additional preliminary evidence suggesting intraspecific variation is extensive in coral hosts and algal symbionts, and that such variation interacts to affect the function of the combined holobiont. The holobiont is both a key ecological feature (being the physical structure that shapes reef ecosystems) and a unit of natural selection; it may ultimately be a unit of evolution in some cases. Future research should incorporate fine-scale molecular genotyping of both partners to address key questions about marine symbiosis ecology and evolution, and to characterize the role of holobiont extended phenotypes in an era of changing climate.

## AUTHOR CONTRIBUTIONS

John E. Parkinson led writing of the manuscript and conducted experiments. Iliana B. Baums formulated the major hypotheses and edited the paper.

## Conflict of Interest Statement

The Guest Associate Editor, Monica Medina, declares that, despite being affiliated with the same institution as authors John E. Parkinson and Iliana B. Baums, the review process was handled objectively and no conflict of interest exists. The authors declare that the research was conducted in the absence of any commercial or financial relationships that could be construed as a potential conflict of interest.
